# Transition to telehealth: Engaging medical students in telemedicine healthcare delivery

**DOI:** 10.1007/s11845-021-02720-1

**Published:** 2021-10-09

**Authors:** Chelsea Cheng, Hilary Humphreys, Bridget Kane

**Affiliations:** 1 School of Medicine, Royal College of Surgeons in Ireland, Dublin, Ireland; 2 Department of Clinical Microbiology, Royal College of Surgeons in Ireland, Dublin, Ireland; 3 Department of Microbiology, Beaumont Hospital, Dublin, Ireland; 4 Karlstad University Business School, Karlstad, Sweden; 5 Centre for Health Policy, Management, Trinity College Dublin, Dublin, Ireland

**Keywords:** COVID-19, Medical education, Medical students, Telehealth, Telemedicine

## Abstract

In recent years, telemedicine has been increasingly incorporated into medical practice, a process which has now been accelerated by the COVID-19 pandemic. As telemedicine continues to progress, it is necessary for medical institutions to incorporate telemedicine into their curricula, and to provide students with the necessary skills and experience to effectively carry out telemedicine consultations. The purposes of this study are to review the involvement of medical students with telemedicine and to determine both the benefits and the challenges experienced. A literature review on the MEDLINE; CINAHL Plus; APA PsychInfo; Library, Information Science and Technology Abstracts; and Health Business Elite databases was performed on September 7, 2020, yielding 561 results. 33 manuscripts were analysed, with the main benefits and challenges experienced by medical students summarized. In addition to increasing their understanding of the importance of telemedicine and the acquisition of telemedicine-specific skills, students may use telemedicine to act as a valuable workforce during the COVID-19 pandemic. Challenges that students face, such as discomfort with carrying out telemedicine consults and building rapport with patients, may be addressed through the incorporation of telemedicine teaching into the medical curricula through experiential learning. However, other more systemic challenges, such as technical difficulties and cost, need to be examined for the full benefits of telemedicine to be realized. Telemedicine is here to stay and has proven its worth during the COVID-19 pandemic, with medical students embracing its potential in assisting in medical clinics, simulation of clinical placements, and online classrooms.

## Introduction

With the rise in telehealth use in recent years and the current rapid progression of the COVID-19 pandemic, the demand for telemedicine is now greater than ever. Telemedicine, as defined by the World Health Organization (WHO), is “The delivery of health care services, where distance is a critical factor, by all health care professionals using information and communication technologies for the exchange of valid information for diagnosis, treatment and prevention of disease and injuries, research and evaluation, and for the continuing education of health care providers, all in the interests of advancing the health of individuals and their communities” [33, p. 10]. Telemedicine is likely to continue growing and become more prevalent in medical care, especially due to the pressures placed on the healthcare system by the COVID-19 pandemic [1, 2, 16, 20, 22, 25]. Telemedicine affords many benefits to patients and care providers alike, for example, improved access to care, lower healthcare costs, and reduced risk of infection for both patients and healthcare providers [9, 18, 25, 28]. As medical students progress in their careers, they will undoubtedly encounter telemedicine in their practice. It is therefore necessary for medical students to develop the skillset needed to effectively deliver patient care through telehealth methods. However, there is a lack of telemedicine teaching and learning within the medical school curricula and telehealth delivery opportunities for medical students [22, 29, 30]. Therefore, this paper reviews the medical student experience with telemedicine to determine the various benefits and challenges of involving medical students with telemedicine, with implications on medical curricula development and future healthcare practices. Although telemedicine includes many domains, this paper focuses specifically on telemedicine as a means of healthcare delivery and the incorporation of telemedicine teaching into medical curricula.

## Methods

A literature review was performed. This section reports on the inclusion and exclusion criteria, as well as the search strategy.

### Study inclusion criteria

All study designs, including clinical studies, review articles, conference abstracts, letters to the editor, and perspectives were included.

- Participants: This review included medical students in any year and stage of training.
- Interventions: Articles discussing the delivery of healthcare through telemedicine in any setting and the incorporation of telemedicine teaching and learning in medical school curricula were included. Telephone consultations were also included.
- Publication date: No time or date restrictions were applied
- Language: Studies in any language were included.

### Study exclusion criteria

Articles that focused on physicians or non-medical students, such as nursing, dental, and veterinary students were excluded. This review also excluded articles that examined application development, continuing medical education, delivery of medical education or examinations, tele-interviews, teleconferences, tele-mentoring, and tele-guidance.

### Literature search strategy

The literature search was performed in the Medical Literature Analysis and Retrieval System Online (MEDLINE); Cumulative Index to Nursing and Allied Health Literature (CINAHL); APA PsychInfo; Library, Information Science and Technology Abstracts; Health Business Elite; and Excerpta Medica Database (EMBASE) databases up to September 7, 2020. The search strategy used the following algorithm: (“medical students” OR “medicine students” OR “students in medicine” OR “student doctors”) AND (“telemedicine” OR “telehealth” OR “telecare” OR “telemonitor”).

## Results and discussion

**Fig. 1:**
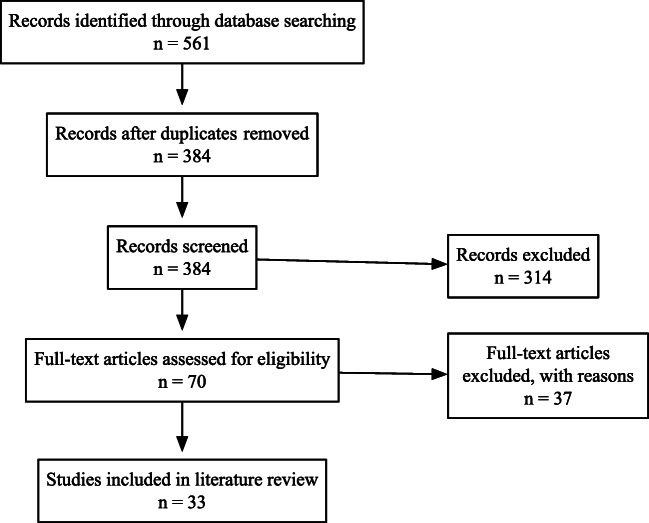
Flow of information through the phases of this review

A total of 561 records were obtained, with 164 from MEDLINE, 61 from CINAHL Plus, 52 from APA PsychInfo, 16 from Library, Information Science and Technology Abstracts, 4 from Health Business Elite, and 264 from EMBASE. Figure 1 is a flow diagram of the phases of the review. Following deduplication, 384 records remained for titles and abstracts screening (Fig. 1). After titles and abstracts screening, there were 70 articles left for full-text screening, following which, a total of 33 articles were included in the review for thematic analysis (Fig. 1). The included articles were analysed by two of the authors independently, and the results were reviewed by the third author. Detailed analyses of the benefits and challenges of involving medical students in telemedicine initiatives were performed and the articles were organized into one of three categories:

- Category 1: Studies evaluating telemedicine theory education with associated clinical telemedicine healthcare delivery (Tables 1 and 2)
- Category 2: Studies solely evaluating clinical telemedicine healthcare delivery (Tables 3, 4, 5 and 6)
- Category 3: Studies solely evaluating telemedicine theory education (Tables 7 and 8)

**Table 1:** Category 1: Studies evaluating telemedicine theory education with associated clinical telemedicine healthcare delivery, continued in next Table

Article	Objectives	Method	Population	Major Findings	Limitations
Abraham, HN et al [1]	– a clerkship for students during COVID-19	Cross-sectional qualitative study	20 third-year medical students at Medical Centre, USA	– Provided diverse clinical experiences	No data on patient satisfaction with model
	– increase student skill & confidence in telehealth with online modules			– Improved student confidence & appreciation of telehealth	
	– participate in weekly telehealth clinic			– High student satisfaction	
				– Learned to adapt to overcome challenges	
Bulik, RJ & GS Shokar [7]	To implement & evaluate a primary care telemedicine elective for fourth-year medical students	Qualitative study	7 fourth-year medical students at US University	– Telemedicine elective a valuable experience	Participation in the elective was self-determined
				– “Very educational & inspiring”	
				– “Look forward to incorporating electronic health into my medical practice”	
				– “The advantages of telemedicine to the field of pathology in particular far outweigh any disadvantages”	
Holubová, A et al [15]	To give medical students insight into current diabetes care technologies	Cross-sectional study	28 medical students at University, Czech Republic	77% evaluated the practical part as very suitable to be included in lectures	Limited results available
Iancu, AM et al [16]	To discuss telehealth opportunities for medical students during & beyond COVID-19	Viewpoint	Medical Students	– Telemedicine skills eg communication, physical exam, professionalism, technological literacy, should be incorporated into medical school curricula	
				– Education through clinical e-visits & virtual consults	
				– Difficulties: Technical challenges, patient discomfort, impaired provider-patient relationships	
Knight, P et al [18]	To support the use of telehealth consultations for medical education	Mixed methods prospective cohort study	2 medical student cohorts (n = 74, n = 76) at Australian University	- Respondents agreed/strongly agreed that the interpersonal aspects were satisfactory	– Short study timeframe
				– Educational opportunities specific to mental health consulting	– Financial incentive payments through Medicare
				– Improved job readiness	
				– Clinical benefits: Continuity of care, timely access to specialists, quicker referrals & follow-ups	
				– Poor picture & sound quality

**Table 2:** Category 1 continued: Studies evaluating telemedicine theory education with associated clinical telemedicine healthcare delivery

Article	Objectives	Method	Population	Major Findings	Limitations
Pathipati, AS et al [22]	To propose a model for incorporation of telemedicine training into medical school curricula	Viewpoint	Medical Students in all years	– Formal telemedicine training should be incorporated into medical schools	
				– Consider “digital call” & “digital health rotations”	
				– Challenges include carrying out physical exams, technology (blood pressure), pain management	
Rolak, S. et al. [25]	To discuss current challenges in medical student education due to COVID-19, responses that have been adopted, and future directions	Opinion review	Medical students in all years	– Benefits: reduces face-to-face contact, reduce risk for viral transmission & need for travel	– Small sample size
				– Can help with routine outpatient telemedicine visits & patient education	Not blinded
				– Challenges include administrative barriers, payment discrepancies	
				– Future suggestions include supervised telemedicine visits, curriculum on virtual etiquette & remote physical exam skills	
Shawagfeh, A. & E. Shanina [27]	To develop a formal Tele-Neurology training program for medical students during their neurology Clerkship	Pilot study	97 medical students (41 fourth year, 56 third year) at US University	– Gained knowledge on telemedicine basics & principles	
				– Improved interprofessional communication, ability to interpret common tests & explain them to the patient	
				– Biggest challenge is taking initiative & providing orientation	
Waseh, S. & A. P. Dicker [30]	To discuss current experiences & learnings from medical schools implementing telemedicine into medical education	Mixed methods review	9 texts meeting inclusion criteria; 70 institutions	– > 60 allopathic medical schools in the USA provide some form of telemedicine experience in their clerkship offerings	- Small sample size
				– 53% (9/17) medical schools utilize patient encounters to develop telemedicine competencies in medical students	– Need further studies on efficacy of telemedicine implementation
Yaghobian, S., et al. [34]	To examine the knowledge, attitudes, and practices of telemedicine education and training	Cross-sectional study	3312 medical students (69.8%) and residents in France	– > 50% felt telemedicine could improve medical practice	- Low participation rates
				– 82.8% believed telemedicine improved access to care	– Overrepresentation of informed students (participation bias)
				– 84.7% who had not practised telemedicine wanted to before the end of their studies

**Table 3:** Category 2: Studies solely evaluating clinical telemedicine healthcare delivery, continued in the next Table

Article	Objectives	Method	Population	Major Findings	Limitations
Annis, T et al. [2]	To evaluate a remote patient monitoring system for patients with COVID-19 symptoms	Mixed methods cross-sectional study	300 patients with COVID-19 symptoms in the USA	– Developed an effective COVID-19 remote monitoring pathway staffed by medical students, residents, & supervising physicians	Requires stronger analysis of patient experience & factors influencing patient participation in pathway
				– High patient satisfaction	
Berwick, KL & L Applebee [5]	To discuss concerns relating to telephone consultations and how these may lessen the positive impacts of placements in general practice	Letter to the editor	Fourth-year medical students at University in England	– Telephone consultations limit opportunities to elicit full histories & perform physical exams	
				– Lifestyle advice less frequently provided, restricting holistic approach to patient management	
Chao, TN et al. [8]	To develop a virtual surgical rotation curriculum during COVID-19	Implement virtual elective	Medical students enrolled in virtual Otolaryngology - Head and Neck Surgery elective at US University	– Provided direct patient contact	– Limited evaluation of medical students’ non-cognitive domains
				– One-on-one engagement with attendings & faculty	– Not a true replacement for clinic & OR experience
				– Exposure to telehealth	– Limited acquisition of exam, procedural & technical skills
Chen, P et al. [9]	Determine attitudes & telehealth use in China among medical professionals & patients; identify factors affecting use	Cross-sectional study	Medical professionals, medical students & patients at 3 large hospitals in China	– 86.91% agree telehealth offered “prompt engagement in self-care”	Respondents all literate; excluded those with little education
				– 72% agree telehealth “reduces healthcare costs”	
				– Concerns: data reliability, privacy, fragmentation of care

**Table 4:** Category 2 continued: Studies solely evaluating clinical telemedicine healthcare delivery, continued in the next Table

Article	Objectives	Method	Population	Major Findings	Limitations
de Araújo Novaes, M, et al. [3]	To improve medical student education through telehealth	Experiment field study	9 medical students in Brazil	– High satisfaction	Data not provided
				– Enriched learning	
				– Increased collaboration among students, faculty & staff	
				– Learned skills necessary for future digital practices	
Dzara, K, et al. [10]	To evaluate training, supervision & usefulness of telepsychiatry rotation	Cross-sectional study	8 residents & 7 medical students in the USA	– 100% agree telepsychiatry enhances overall training	- Small sample size
				– 86.7% believe telepsychiatry can treat variety of conditions	– No objective pre-/post-test
				– 60% had technical difficulties	– No validated survey tool
				– 60% had difficulty reading patient affect	– Did not assess if didactic or clinical preparation influenced views
				– 64.3% wanted more clinical experience	
				– 80% felt more comfortable using telepsychiatry in the future	
Greisman, L., et al. [11].	To evaluate feasibility & cost of a smartphone-based teledermatology consult service	Cross-sectional study	2 fourth-year medical students in the USA; 93 cases evaluated by the consult service	– In 100% of cases, medical students provided enough information for diagnosis	- Small sample size
				– Students became familiar with specific management recommendations	– Limited sustainability
				– Collaborative team approach	
				– Poor patient follow-up	
				– Fixed upfront cost	
				– Students wanted greater supervision	
Heflin, KJ, et al. [12]	To describe a medical student-led initiative to provide care during COVID-19	Editorial/Case study	16 medical students in the USA	– Tele-health services may have improved continuity of care	Lack of access to telehealth for individuals dealing with homelessness
				– Facilitated conversations about social determinants of health	
				– Patients no longer wished to continue participating once health needs met	
				– Technical and access challenges

**Table 5:** Category 2 continued: Studies solely evaluating clinical telemedicine healthcare delivery, continued in the next Table

Article	Objectives	Method	Population	Major Findings	Limitations
Himstead, AS, et al. [13].	To determine if medical students could sufficiently use a non-mydriatic fundus camera in a teleophthalmology program	Retrospective study	Medical students participating in Floating Doctors Non-profit Org. in Panama; 126 images from 70 patients	– Most common (37.07%) photo quality was “Not ideal, but able to exclude subtle findings”	Limited results available
				– With minimal training, medical students & GPs can use remote retinal cameras to assist with diagnosis & treatment of ophthalmic conditions	
Mukundan, S, Jr, et al. [19].	To create a telemedicine system for medical students on elective study at remote locations	Experiment	1 senior visiting British medical student; Telemedicine system between Solomon Islands & USA	– Telemedicine system provided considerable support to physicians & patients at each remote location	Full data not obtained for all referrals
				– Allowed faculty at central site to continue to supervise students abroad	
Rallis, KS & AM Allen Tejerina [23]	To discuss integration & challenges of telemedicine education & training in tele-oncology	Letter	Medical students	– Suggestions: streaming tele-oncology clinics, virtual MDTs, remote access to electronic medical records	
				– Challenges: difficulty practising physical exams, empathy, motivational interviewing, patient discomfort	
Schmidt, S & E Sheline [26]	To describe an telehealth coaching pilot for medical students to teach patient self-management skills	Cross-sectional study	30+ medical students at Emory University, USA	– High learner satisfaction - impactful experiences & improved understanding of barriers patients face in accessing health care	– Small sample size
				– Positive clinical outcomes for patients - 73% of patients who attended more than one Healthy Living class lost weight	– No control group
					– Selection bias

**Table 6:** Category 2 continued: Studies solely evaluating clinical telemedicine healthcare delivery

Article	Objectives	Method	Population	Major Findings	Limitations
Vasquez-Cevallos, LA, et al. [28]	To present Telemedicine Platform (TMP) for rural healthcare services in Ecuado	Field study	124 senior undergraduate medical students (63 completed questionnaire) & 6 faculty members in Ecuador; 262 teleconsultations	– Telemedicine Platform useful for learning (100%)	– Small sample size
				– Advantages: increased access to specialists, enhanced practical knowledge, assistance in diagnosis & treatment	– Different student numbers in each period
				– 100% would use the platform in their rural service	
				– Challenges: delays by faculty in answering, connectivity problems	
Wernhart, A, et al. [29]	To assess how medical students & healthcare professionals perceive eHealth & telemedicine	Cross-sectional study	51.6% medical students in Austria	– Participants expressed moderate knowledge of eHealth & telemedicine concepts; higher levels among employees compared to students	– Self-reported data (response bias)
				– Students optimistic that telemedicine reduces healthcare costs	– Sampling limits generalizability
				– Doubts if telemedicine enhances doctor-patient relationship	
				– Data security & privacy issues	
Whittemore, MS, et al. [32]	To evaluate an insulin titration telemedicine program	Cross-sectional study	Medical students, volunteer endocrinologists & diabetes educators in the USA	– Significant decrease found between pre- & post-intervention HbA1c’s	– Limited results available
					– Further evaluation needed

**Table 7:** Category 3: Studies solely evaluating telemedicine theory education, continued in the next Table

Article	Objectives	Method	Population	Major Findings	Limitations
Barth, J, et al. [4]	To document & identify reasons for insecurity in medical students during emergency phone consultations	Mixed methods cross-sectional cohorts	137 fifth-year medical students from Inst of Family Medicine, Switzerland	– Students felt most insecure during history-taking due to lack of clinical knowledge & experience, urgency of call	Limited generalizability
				– Doubts about reliability of information from caller	
				– Lack of physical examination	
Brockes, C, et al. [6]	To systematically evaluate “Clinical Telemedicine/e-Health” module over 8 years	Mixed methods prospective cohorts	Second-, third- & fourth-year medical students in Switzerland (23–35 students in various years)	– In 2015, 93% of students wanted to provide telemedicine care for chronic and older patients in their homes	– Evaluations were not performed in the same way every year
				– Increased overall satisfaction & understanding of telemedicine as a supplement in traditional medical consultations	– Number of participating students changed year-by-year
Jonas, CE, et al. [17]	To design, administer & evaluate an Introduction to Telehealth course	Cross-sectional study	149 third-year medical students (uniformed services) in the USA	– High interest and acceptance of course	Limited generalizability (unique study pop.)
				– 10.1% increase in knowledge is modest for time invested	
				– 80% indicated future plans to practice telehealth	
Hindman, D. J., et al. [14].	To report on implementation of a telephone medicine curriculum (part of paediatrics clerkship)	Prospective cohort study	245 medical students, with 67 students receiving the intervention	– Students who received the telephone medicine curriculum had significantly higher mean overall scores on simulated OSCE telephone medicine case	– Confounding - some students completed paediatrics clerkship prior to study
					– Convenience sampling
					– Limited generalizability

**Table 8:** Category 3 continued: Studies solely evaluating telemedicine theory education

Article	Objectives	Method	Population	Major Findings	Limitations
Naik, N, et al. [20]	To develop a simulation program for video-based communication skills	Cross-sectional study	Fourth-year medical students in the USA	– Initial feedback for the course was positive	– Limited results available
				– Taught students telemedicine-specific communication skills	– Further evaluation needed
Newcomb, AB, et al. [21]	To pilot a class to improve communication skills in video consults	Cross-sectional study	5 fourth-year medical students in a surgical internship, USA	– Felt the class introduced new skills & reinforced current ones	Small sample size limiting generalizability
				– Most reported higher self-confidence in target communication skills following the module	
				– Difficulty interpreting patient distress	
				– Poor lighting and body positioning	
Rienits, H, et al. [24]	To develop a clinical skills lesson to prepare students for rural practice placements	Mixed methods cross-sectional study	59 third-year medical students	– Improved understanding of the issues, procedures & confidence in conducting a telehealth consultation	Small sample size
Walker, C, et al. [29]	To introduce an educational intervention to improve student knowledge and confidence with telemedicine	Cross-sectional study	153 second-year medical students (93 completed questionnaire)	– Higher mean post-test scores in telemedicine knowledge & confidence	– Lack of objective measures
				– Most improvement in equipment operation & “designing an office conducive for a telemedicine visit”	– Low response rate
				– Students want more simulation time	

The studies involving medical students in telemedicine initiatives were diverse and spanned studies with fewer than thirty students in educational initiatives [6], to a study with over 3300 students involved with telemedicine practice in a clinical setting [34]. Of the 33 articles included in this review, two experimental design controlled studies were identified [4, 14] (Table 7).

### Benefits of involving medical students in telemedicine

Involving medical students in telemedicine resulted in positive outcomes for students, staff, and patients alike. Medical students who participated in telemedicine practice found the experience valuable [1, 7] and reported improved knowledge and understanding of telemedicine [1, 3, 7, 8, 10, 17–19, 24, 27, 29]. In the study by Abraham et al., 87% of students strongly agreed that telehealth would be “a valuable service to offer to patients” [1, p. 4]. As well, students gained a better understanding of the use of telemedicine as an adjunct to traditional medical consultations [6], and believed that it would play an important role in their future careers [1, 7, 10, 17, 28]. Jonas et al. found that 80% of surveyed students responded that they would like to incorporate telemedicine into their future practices [17]. By participating in telemedicine curricula, students learned the skills needed to effectively carry out telemedicine consults [1, 3, 17, 20, 21, 24, 27, 29], such as communicating empathy [1, 21] and equipment set-up and use [17, 20]. Moreover, students who attended a virtual class as part of a surgical internship reported higher self-confidence levels in telemedicine-specific communication domains such as exploring patient’s perceptions and concerns [21].

Students who participated in healthcare delivery using telemedicine appreciated the role of telemedicine in various specialties [3, 7, 10, 13, 18, 27], including its use in General Practice [18], Psychiatry [10], Neurology [27], and Ophthalmology [13]. Medical and surgical specialities that currently already utilize telemedicine in their practice, in particular the Primary Care speciality [1, 5, 7, 12, 18, 28, 32], may benefit further from involving students in clinical telemedicine healthcare delivery and telemedicine education initiatives (Table 9).

**Table 9:** Medical and surgical subspecialties using telemedicine with medical students

Article	Specialty
Abraham, H. N., et al.	Primary Care
Berwick, K. L. and L. Applebee	Primary Care
Bulik, R. J. and G. S. Shokar	Primary Care
Heflin, K. J., et al.	Primary Care; Mental Health
Knight, P., et al.	Primary Care
Vasquez-Cevallos, L. A., et al.	Primary Care
Whittemore, M. S., et al.	Primary Care
Shawagfeh, A. and E. Shanina	Neurology
Rallis, K. S. and A. M. Allen Tejerina	Oncology
Dzara, K., et al.	Psychiatry
Greisman, L., et al.	Dermatology
Himstead, A. S., et al.	Ophthalmology
Chao, T. N., et al.	Otolaryngology

Telemedicine also provided an opportunity for increased collaboration among students, faculty and staff [3, 8, 11, 18, 19, 27], through opportunities to have more one-on-one time with faculty members [8] and the chance to observe specialists working with general practitioners [18]. Students enjoyed being exposed to a variety of acute and chronic patient cases [1, 3] and having the opportunity to increase their medical knowledge [18, 28]. For example, students learned how to interpret common test results [27] and became familiar with specific management recommendations for skin conditions such as eczema and filariasis [11].

Engaging students in telemedicine also resulted in positive patient outcomes [11, 12, 18, 19, 26, 28, 32]. Through telemedicine, patients could be offered comprehensive care and access to specialists [18, 28]. Telemedicine initiatives may also improve patient self-efficacy, for example, in a study by Schmidt and Sheline, 73% of patients who participated in a student-run “Healthy Living” telehealth coaching program lost weight successfully [26]. Studies by Himstead et al. and Mukundan et al. also reported good patient outcomes with minimal student training [13, 19]. Students developed a deeper understanding of patient barriers in accessing healthcare [26] and social determinants of health [12]. Students acknowledged that telemedicine would be particularly beneficial for vulnerable populations, such as the elderly or those with chronic conditions, as care could be provided in their homes [6]. In Whittemore et al.’s study, patients with diabetes mellitus who participated for three or more months in an insulin titration telemedicine program staffed by medical students, endocrinologists, and educators, were found to have significantly lower HbA1c levels [32].

### Challenges of involving medical students in telemedicine

Despite the many benefits that medical students experienced when engaging with telemedicine, there were also challenges. Student-specific challenges included a range of comfort with completing telemedicine visits [1, 4], which may have been due to lack of clinical knowledge and experience [4], and concern about their ability to build rapport and convey empathy [1, 23]. For the latter, students found timely feedback and demonstrations from supervising doctors beneficial in improving their communication skills [1]. Another challenge that frequently arose with telemedicine consults, especially in telephone consults, was the lack of visual impression and ability to perform a physical examination [4, 5, 22, 23]. Moreover, students found it difficult to read patients’ affect [10] and interpret patient distress [21]. In a letter to the editor, fourth-year medical students drew attention to difficulties obtaining full histories from patients when using telemedicine, and that when compared with traditional medical consults, the explanation of diagnoses, treatment, and lifestyle advice was less frequently offered to patients [5]. Students questioned the reliability of information provided by callers [4, 9] and some noted poor patient follow-up [11]. Students from the Medical University of Vienna expressed doubts that telemedicine could enhance the doctor-patient relationship [31]. Another patient concern was privacy and data security [9, 31].

Although telemedicine provided opportunities for learning, Chao et al. described educational barriers such as a lack of exposure to the full scope of practice, difficulties in acquiring procedural skills, and limitations in evaluating students on non-cognitive domains [8].

In terms of environmental challenges, students experienced technical difficulties such as connectivity issues and poor image and sound quality [1, 10, 13, 18, 28]. Although patients often faced challenges with technology, some students enjoyed the educational opportunity to assist patients with using telehealth platforms [1]. There were also cost discrepancies in telemedicine usage [25], with some reporting a fixed upfront cost [11], while others believed telemedicine reduced costs [9, 31].

### Telemedicine during COVID-19

Six of the studies were conducted in the context of the COVID-19 pandemic in clinical settings [1, 2, 8, 12, 16, 25] and three of these did not include an education component [2, 8, 12].

Students appreciated the opportunity to engage in patient care during the COVID-19 pandemic through telemedicine initiatives [1, 2, 8]. For instance, in one COVID-19 virtual health rotation, medical students provided a valuable workforce in terms of staffing a remote patient monitoring pathway [2]. However, a big challenge was adjusting the number of staff to match demand, as the number of enrolled patients changed over time [2]. Further engaging medical students with this pathway may allow discrepancies to be addressed. The COVID-19 pandemic has accelerated the delivery of remote clinical education via telemedicine [16], while reducing the risk for COVID-19 transmission [25]. There was also a role for students to help with routine outpatient telemedicine visits during the pandemic [25]. A free clinic run by medical students to serve homeless individuals during COVID-19 saw improved continuity of care that could circumvent lack of access to hospitals and transportation; however, an issue that arose was that homeless patients no longer wanted to continue participating once their health needs were met [12]. In spite of some challenges, telemedicine may offer a safe and viable method for healthcare professionals, including medical students, to continue delivering quality healthcare to patients during the COVID-19 pandemic.

### Incorporating telemedicine into medical curricula

Telemedicine has been successfully introduced into medical curricula at a number of institutions worldwide, including in the USA, Switzerland, Brazil, the Czech Republic, and Australia [1, 3, 6–8, 10, 14, 15, 17, 18, 20–22, 24, 26, 27, 29, 30], and its use should be more widely adopted. There is a large demand for more telemedicine opportunities, with students displaying high interest and support for its incorporation into their learning [7, 15, 34]. To facilitate the development of a telemedicine curricula, medical institutions should consider making improvements to existing programs. For instance, Iancu et al. suggested incorporating telemedicine-specific skills, such as communication that minimizes body motions, functional physical examination skills, and technological literacy, into medical curricula [16]. Pathipati et al. recommended a digital call experience which would allow students to familiarize themselves with remote monitoring tools and participate in consultations [22]. Students who participated in telemedicine programs wished for more clinical experiences [10], such as utilizing telemedicine in clinical practice [30]. They also hoped for more simulation time [29] and greater supervision [11, 25]. This student feedback should be taken into account when developing future curricula. With COVID-19 continuing to disrupt medical education and person-to-person contact, telemedicine provides a feasible alternative to maximize clinical exposure for students and to experience a technology that is here to stay. Medical institutions should capitalize on this opportunity to update medical curricula in keeping with the technological advances occurring in telemedicine and beyond.

### Strengths and limitations

The strengths of this literature review are that there are virtually no limitations to the search criteria, such as being confined to the English language, time period constraints, or restriction in study design. Thus, the depth of experiences conveyed and the perspectives of the students’ themselves are all considered. This study supplements existing literature by identifying and summarizing key benefits and considerations in medical students’ experiences with telemedicine. Limitations of this study include the diversity and heterogeneous nature of the papers necessitating a literature review rather than a systematic review and that the COVID-19 is ongoing, hence, this subject is a rapidly changing landscape.

## Conclusion

This literature review summarized medical students’ experiences with telemedicine, in an effort to determine its benefits and challenges. Telemedicine offers a valuable clinical experience for medical students, providing knowledge and skills that they could apply in their future practices. In addition, it provides students with opportunities to collaborate with other faculty and staff, and the ability to continue with clinical learning during the COVID-19 pandemic. Despite the benefits, several challenges were also identified, such as difficulty in conveying empathy and problems with technology. However, with medicine becoming increasingly technologized and the COVID-19 pandemic continuing to disrupt medical education, now is the time for medical institutions to consider incorporating telemedicine teaching and learning into the curricula, so that the medical students of today will be prepared for the medical practice of tomorrow.
